# Allergic reactions to tick saliva components in zebrafish model

**DOI:** 10.1186/s13071-023-05874-2

**Published:** 2023-07-19

**Authors:** Marinela Contreras, Rita Vaz-Rodrigues, Lorena Mazuecos, Margarita Villar, Sara Artigas-Jerónimo, Almudena González-García, Nadezhda V. Shilova, Nicolai V. Bovin, Sandra Díaz-Sánchez, Elisa Ferreras-Colino, Iván Pacheco, Jindřich Chmelař, Petr Kopáček, Alejandro Cabezas-Cruz, Christian Gortázar, José de la Fuente

**Affiliations:** 1 SaBio, Instituto de Investigación en Recursos Cinegéticos IREC-CSIC-UCLM-JCCM, Ronda de Toledo s/n, 13005 Ciudad Real, Spain; 2 Biochemistry Section, Faculty of Sciences and Chemical Technologies, Universidad de Castilla-La Mancha, Ave. Camilo José Cela 10, 13071 Ciudad Real, Spain; 3 Shemyakin-Ovchinnikov Institute of Bioorganic Chemistry, Russian Academy of Sciences, Miklukho-Maklaya str. 16/10, 117997 Moscow, Russian Federation; 4 National Medical Research Center for Obstetrics, Gynecology and Perinatology Named After Academician V. I. Kulakov, Oparina str. 4, 117198 Moscow, Russian Federation; 5 Centre for Kode Technology Innovation, School of Engineering, Computer and Mathematical Sciences, Faculty of Design and Creative Technologies, Auckland University of Technology, Auckland, New Zealand; 6 Department of Medical Biology, Faculty of Science, University of South Bohemia in České Budějovice, Branišovská 31, 37005 České Budějovice, Czechia; 7 Institute of ParasitologyBiology Centre, Czech Academy of Sciences, Branišovská 31, 37005 České Budějovice, Czechia; 8 UMR BIPAR, INRAE, ANSES, Ecole Nationale Vétérinaire d’Alfort, Université Paris-Est, 94700 Maisons-Alfort, France; 9 Department of Veterinary Pathobiology, Center for Veterinary Health Sciences, Oklahoma State University, Stillwater, OK 74078 USA; 10 Present Address: Departamento de Bioquímica, Microbiología, Biología Celular y Genética, Área de Microbiología, Universidad de La Laguna, Entrada Campus Anchieta, 4, 38200 La Laguna, Tenerife, Canary Islands Spain

**Keywords:** Allergy, Alpha-gal syndrome, Glycan, Tick, Zebrafish

## Abstract

**Background:**

Alpha-Gal syndrome (AGS) is a tick-borne food allergy caused by IgE antibodies against the glycan galactose-alpha-1,3-galactose (α-Gal) present in glycoproteins and glycolipids from mammalian meat. To advance in the diagnosis and treatment of AGS, further research is needed to unravel the molecular and immune mechanisms underlying this syndrome. The objective of this study is the characterization of tick salivary components and proteins with and without α-Gal modifications involved in modulating human immune response against this carbohydrate.

**Methods:**

Protein and α-Gal content were determined in tick saliva components, and proteins were identified by proteomics analysis of tick saliva fractions. Pathophysiological changes were recorded in the zebrafish (*Danio rerio*) model after exposure to distinct *Ixodes ricinus* tick salivary components. Serum samples were collected from zebrafish at day 8 of exposure to determine anti-α-Gal, anti-glycan, and anti-tick saliva protein IgM antibody titers by enzyme-linked immunosorbent assay (ELISA).

**Results:**

Zebrafish treated with tick saliva and saliva protein fractions combined with non-protein fractions demonstrated significantly higher incidence of hemorrhagic type allergic reactions, abnormal behavioral patterns, or mortality when compared to the phosphate-buffered saline (PBS)-treated control group. The main tick salivary proteins identified in these fractions with possible functional implication in AGS were the secreted protein B7P208-salivary antigen p23 and metalloproteases. Anti-α-Gal and anti-tick salivary gland IgM antibody titers were significantly higher in distinct saliva protein fractions and deglycosylated saliva group when compared with PBS-treated controls. Anti-glycan antibodies showed group-related profiles.

**Conclusions:**

Results support the hypothesis that tick salivary biomolecules with and without α-Gal modifications are involved in modulating immune response against this carbohydrate.

**Graphical Abstract:**

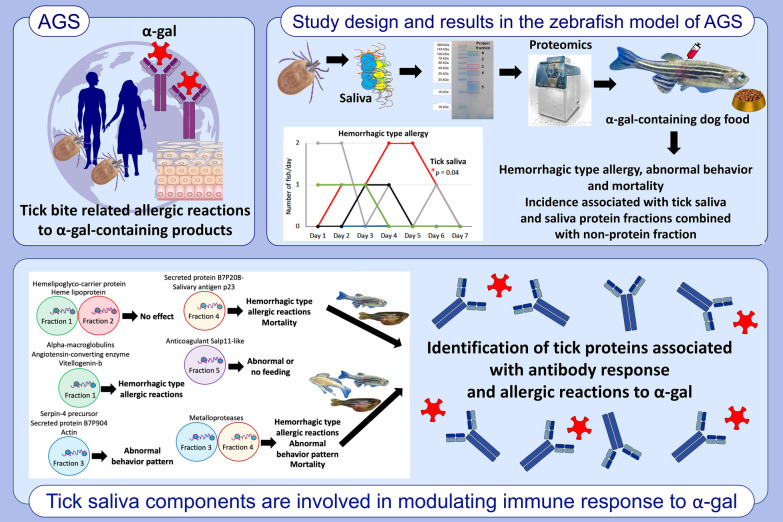

**Supplementary Information:**

The online version contains supplementary material available at 10.1186/s13071-023-05874-2.

## Background

Alpha-Gal syndrome (AGS), also known as mammalian meat allergy, is a tick-borne allergy caused by immunoglobulin E (IgE) response to glycan galactose-alpha-1,3-galactose (α-Gal) modification of protein and lipid glycoconjugates [1–6]. The initial IgE sensitization is linked to bites from hard-bodied ticks such as the castor bean tick *Ixodes ricinus* in Europe and the lone star tick *Amblyomma americanum* in North America [7, 8]. Clinical presentation comprises delayed hypersensitivity to the consumption of non-catarrhine mammalian meat and its derivatives and immediate-onset anaphylaxis to α-Gal-containing drugs (mammalian-based substances), likely because drugs are parenterally administered and not consumed [9, 10].

The tick saliva immunogenic agents and triggering pathway of AGS have not yet been totally revealed. AGS patients typically show a Th2-skewed profile with increased anti-α-Gal IgE and IgG levels and allergen-specific B cells and basophil stimulation [8, 11–14]. Recently, the enzyme α-D-galactosidase has been identified as a regulator of α-Gal production in tick salivary glands [15, 16]. Tick saliva contains various biogenic substances with main components such as water, ions, non-peptide molecules such as glycans, tick and host proteins, and exosomes [17–21].

To advance in the diagnosis, treatment, and prevention of AGS, it is important to address the question of why only some individuals exposed to tick bites develop AGS [22]. In addition to differences in tick α-Gal content [23], there is variability in who will and will not become sensitized, and among individuals who have become sensitized, some become allergic to mammalian meat but others can continue tolerating it. To address this question, we hypothesize that tick salivary components with and without α-Gal modifications are involved in modulating the human immune response against this carbohydrate.

To help address this hypothesis, herein we used the proposed α-Gal-negative zebrafish (*Danio rerio*) animal model for AGS [24]. In a previous study [24], zebrafish treated with tick saliva and fed mammalian meat showed AGS-associated responses such as differential granulocyte profiles with basophils/eosinophils and upregulation of allergic disease biomarkers such as interleukin-1 beta and interleukin-4 [9, 25] also associated with responses to allergens in zebrafish [26] and mice [27]. Furthermore, as recently reviewed [28], zebrafish have been previously used as an animal model for food-associated allergy and immunity [29, 30]. Additionally, when compared to phosphate-buffered saline (PBS)-treated controls, zebrafish treated with tick saliva following mammalian meat consumption but not with any of these components alone presented a higher incidence of wide inter- and intrapersonal variability signs associated with AGS [31] such as hemorrhagic type allergic reactions (urticaria and angioedema in AGS), abnormal behavior (respiratory distress in AGS) and feeding (gastrointestinal symptoms, diarrhea, abdominal pain, reflux, emesis in AGS), and mortality [24]. These findings support the use of a zebrafish animal model to characterize AGS-associated signs in response to tick saliva and mammalian meat consumption.

In this study, zebrafish were exposed to *I. ricinus* tick saliva protein and non-protein components to evaluate the incidence of local hemorrhagic type allergic reactions, altered behavior patterns and feeding, and mortality. The results identified tick saliva proteins as candidate immunoregulatory in combination with non-protein salivary components involved in AGS.

## Methods

### Ethics statement

Experiments in zebrafish were conducted in strict accordance with the recommendations of the European Guide for the Care and Use of Laboratory Animals. Fish were housed and experiments were conducted at an experimental facility (Catalonia Institute for Energy Research [IREC], Ciudad Real, Spain) with the approval and supervision of the Ethics Committee on Animal Experimentation of the University of Castilla La Mancha (PR-2021-09-14) and the Department of Agriculture, Environment and Rural Development of Castilla La Mancha (REGA code ES130340000218).

### Experimental design

**Fig. 1:**
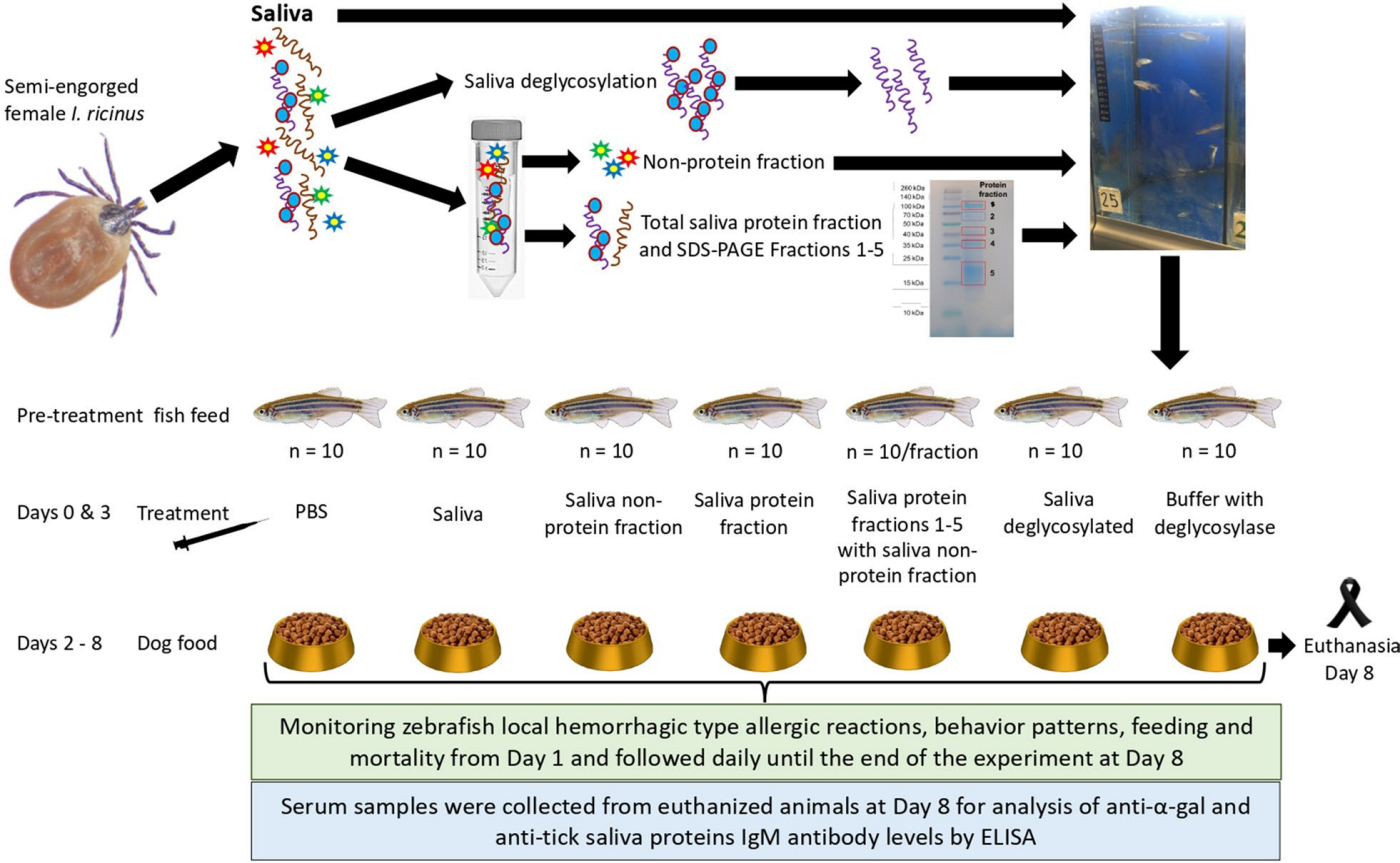
Experimental design to characterize tick saliva components associated with allergic reactions to mammalian meat consumption in the zebrafish model of alpha-Gal syndrome (AGS). Saliva from semi-engorged *Ixodes ricinus* female ticks was collected and used to prepare protein, non-protein, and deglycosylated saliva fractions. Tick saliva fractions with quantified protein content were used for treatment of zebrafish. PBS and buffer with deglycosylase were used as negative controls. Wild-type adult AB strain zebrafish (10 animals per group; 1:1 female to male ratio) were kept on fish feed during pretreatment and until day 2. Zebrafish were injected with each treatment at days 0 and 3, and from day 2 until the end of the experiment at day 8 fish were fed dog food containing mammalian meat. Zebrafish hemorrhagic type allergic reactions, abnormal behavior patterns and abnormal or no feeding, and cumulative mortality were examined after feed change and treatment at day 3 and followed daily until the end of the experiment at day 8. After fish euthanasia, serum was collected from each animal to determine anti-α-Gal and anti-tick saliva protein IgM antibody titers

**Fig. 2:**
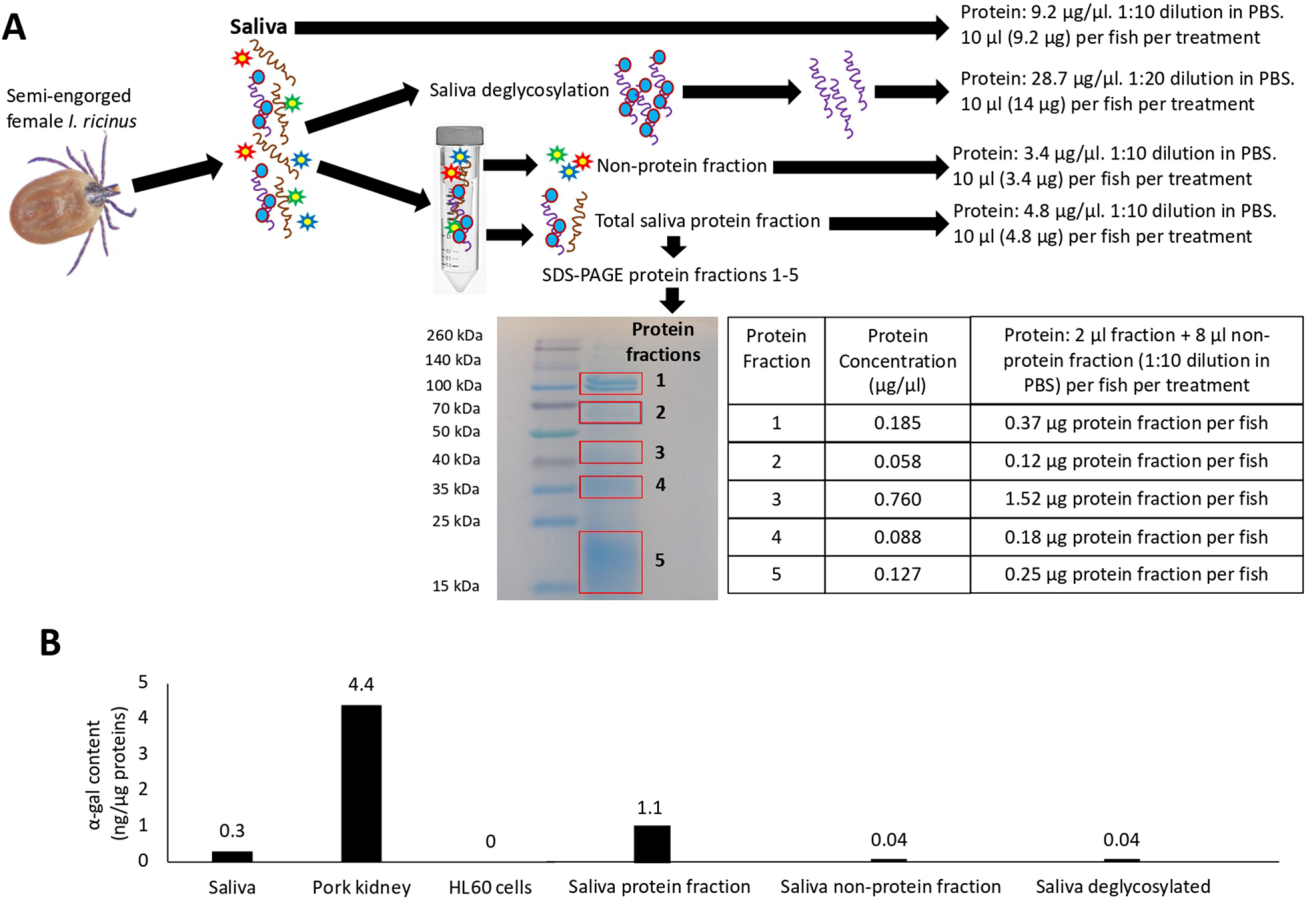
Protein and α-Gal content in tick saliva components and fractions. **A** Saliva from semi-engorged *Ixodes ricinus* female ticks was collected and used to prepare protein, non-protein, and deglycosylated saliva fractions. Protein content was quantified in tick saliva fractions used for treatment of zebrafish. **B** The α-Gal content was quantified by ELISA in tick saliva and tick saliva protein, non-protein, and deglycosylated components in comparison with pig kidney (positive control) and human Caucasian promyelocytic leukemia HL60 cells (negative control). The quantitation of α-Gal content was performed twice, with similar results

The experiment was designed to characterize tick saliva components associated with allergic reactions to mammalian meat consumption in the zebrafish model of AGS (Fig. 1, Ref. [24]) Saliva from semi-engorged *I. ricinus* female ticks was collected and used to prepare protein, non-protein, and deglycosylated fractions. The α-Gal content was quantified in tick saliva in comparison with pig kidney (positive control) and human Caucasian promyelocytic leukemia HL60 cells (negative control) as described previously [24]. Protein content was quantified in tick saliva and its fractions used for treatment of zebrafish (Fig. 2A). The amount of protein administered by fish is shown in Fig. 2A. PBS and buffer with deglycosylase were used as negative controls. Wild-type adult [6–8-month-old) AB strain zebrafish (10 animals per group; 1:1 female-to-male ratio; 330 ± 70 mg weight) were kept on fish feed during pretreatment and until day 2. At days 0 and 3, zebrafish were intramuscularly injected with each treatment, and from day 2 until the end of the experiment at day 8 fish were fed dog food containing mammalian meat. Zebrafish hemorrhagic type allergic reactions (skin redness), behavior (abnormal behavior patterns and abnormal or no feeding), and cumulative mortality were examined throughout the experiment and compared between groups to assess the effect of treatments and dog food after feed change and treatment between days 1 and 7 as reported previously [24]. After fish euthanasia, serum was collected from each animal to determine anti-α-Gal and anti-tick saliva protein IgM antibody titers equivalent to human IgE/IgG antibodies [32]. Kidney and intestine samples were collected from euthanized animals at day 8 and stored at −80 °C for further analysis.

### *Ixodes ricinus* tick saliva

Pathogen-free *I. ricinus* ticks were obtained from the laboratory colony maintained at the Institute of Parasitology, Biology Centre of the Czech Academy of Sciences of the Czech Republic (IPBCAS), in České Budějovice. All animal experiments were performed in accordance with the Animal Protection Law of the Czech Republic no. 246/1992 Sb (ethics approval no. 34/2018). Semi-engorged female ticks fed for 6–7 days on guinea pigs were inoculated into the hemocoel with 5 μl of a 2% (w/v) solution of pilocarpine hydrochloride (Sigma-Aldrich, St. Louis, MO, USA) in PBS, and approximately 0.6 μl of saliva was collected per tick as described previously [24, 33]. Saliva was then transported and stored at −80 °C until use.

### Tick saliva protein and non-protein fractions

Tick saliva (135 µl) was diluted 1:1 in PBS, and 255 µl was filtered twice through an Amicon 3 kDa unit (Merck & Co., Inc., Kenilworth, NJ, USA). Of this, 200 µl passed through the Amicon membrane and was considered the non-protein fraction. The 50 µl that did not pass through the Amicon membrane was considered the protein fraction.

###  Glycosidase treatment of tick saliva

For protein deglycosylation, 20 µl of tick saliva was incubated under denaturing conditions with a cocktail of α-Gal-free glycosidases (PNGase F, 36 kDa; α-(2-3,6,8,9)-neuraminidase, 69 kDa; *O*-glycosidase, 180 kDa; β(1-4)-galactosidase, 350 kDa; β-*N*-acetylglucosaminidase, 140 kDa) that removes both asparagine-linked (*N*-linked) and serine/threonine-linked (*O*-linked) oligosaccharides using the EDEGLY enzymatic protein deglycosylation kit (Merck & Co., Inc.) and following the manufacturer’s recommendations [34]. After deglycosylation, the tick saliva sample was diluted 1:20 in PBS for filtration first through an Amicon 50 kDa unit (Merck & Co., Inc.) to remove deglycosylases except PNGase F, and then the flow-through with tick salivary proteins (most proteins had less than 50 kDa; Fig. 2A) was filtered through the Amicon 3 kDa (Merck & Co., Inc.) to remove buffer and retain proteins.

### Tick saliva protein fractionation by sodium dodecyl sulfate polyacrylamide gel electrophoresis (SDS-PAGE) and characterization by mass spectrometry analysis

To obtain the different tick saliva protein fractions, 70 µg from the saliva protein fraction was mixed in 1:1 proportion with Laemmli sample buffer and applied onto two 1.2-cm-wide wells on two 10% SDS-PAGE gels. In one gel, protein bands were visualized by staining with GelCode Blue Stain Reagent (Thermo Fisher Scientific, Waltham, MA, USA), excised, cut into 2 × 2 mm cubes, and digested overnight at 37 °C with 12.5 ng/μl sequencing grade trypsin (Promega, Madison, WI, USA) at a ratio of 5:1 protein/trypsin (w/w) in 50 mM ammonium bicarbonate, pH 8.8, containing 10% (v/v) acetonitrile [35]. The resulting tryptic peptides from each band were extracted by incubation for 30 min in 12 mM ammonium bicarbonate, pH 8.8. Trifluoroacetic acid was added to a final concentration of 0.1%, and the peptides were finally desalted onto OMIX C18 pipette tips (Agilent Technologies, Santa Clara, CA, USA), dried down, and stored at −20 °C until use for mass spectrometry analysis. The desalted protein digests were resuspended in 0.1% formic acid and analyzed by reversed-phase liquid chromatography coupled to mass spectrometry (RP-LC–MS/MS) using an EASY-nLC II system coupled online to an LTQ Linear Ion Trap mass spectrometer (Thermo Fisher Scientific). The peptides were concentrated using a 0.1× 20 mm C18 reversed-phase (RP) precolumn (Thermo Fisher Scientific) and separated using a 0.075× 100 mm C18 RP column (Thermo Fisher Scientific) operating at 0.3 μl/min. Peptides were eluted using a 60-min gradient from 5 to 40% solvent B in solvent A (solvent A: 0.1% formic acid in water, solvent B: 0.1% formic acid, 80% acetonitrile in water). Electrospray ionization (ESI) was carried out using a nano-bore emitter stainless steel ID 30 μm (Thermo Fisher Scientific) interface. Peptides were detected in survey scans from 400 to 1600 atomic mass units (amu, 1 μscan), followed by 15 data-dependent MS/MS scans (Top 15), using an isolation width of two mass-to-charge ratio units, normalized collision energy of 35%, and dynamic exclusion applied for periods of 30 s. Peptide identification from the MS/MS raw data was carried out using the SEQUEST algorithm (Proteome Discoverer 1.4; Thermo Fisher Scientific). A search was performed against the Ixodidae UniProt protein database (184,796 entries in July 2020). The following constraints were used for the searches: tryptic cleavage after Arg and Lys, up to two missed cleavage sites, and tolerance of 1 Da for precursor ions and 0.8 Da for MS/MS fragment ions, and the search was performed allowing optional methionine oxidation and cysteine carbamidomethylation. A search was performed against a decoy database in an integrated decoy approach. A false discovery rate (FDR) < 0.01 was considered as a condition for successful peptide assignments, and at least two peptides per protein was the condition for successful protein identification. Protein bands from the second gel were excised and cut into small cubes, covered with PBS with 0.1% SDS, and incubated in a rotator overnight at 4 °C. The supernatants containing the protein fractions were methanol/chloroform-precipitated, resuspended in PBS for quantification by bicinchoninic acid (BCA) protein assay kit (Bio-Rad, Hercules, CA, USA), and stored at −20 °C until fish treatment.

### Tick protein annotations

Tick proteins identified after proteomics analysis were annotated for Gene Ontology in UniProt (https://www.uniprot.org) and VectorBase (https://vectorbase.org/vectorbase/app/). Biological processes include annotations in *Drosophila* or human proteins that may be related to AGS when information is not available in tick species. Sequences from all identified proteins were used for Basic Local Alignment Search Tool (BLAST) analysis (UniProtKB reference proteomes plus Swiss-Prot; E-threshold = 10) in UniProt (Table 1, Additional file 1: Dataset S1). Additionally, for the secreted protein B7P208—salivary antigen p23 A0A0K8RKR7 (Table 1, Additional file 1: Dataset S1), match to 3UV1_A Chain(A) PDB structure of allergen from dust mite (https://www.rcsb.org/structure/3UV1) was predicted using PredictProtein (https://predictprotein.org) tool (identity = 0.20, expected value = 1e−28, matched length = 205 of 222 to A0A0K8RKR7) (Table 2, Additional file 1: Dataset S1).

**Table 1:** Protein identification by mass spectrometry in tick saliva fractions. Full data are provided in Additional file 1: Dataset S1

UniProt Protein ID	Protein description	Mass spectrometry data		
		Score	Coverage (%) No. proteins unique peptides PSMs	No. amino acids MW (kDa) Calculated pI
Fraction 1: Hemorrhagic type allergic reactions				
B7Q407	Heme lipoprotein	272.12	21.75, 1, 23, 94	1329, 152.5, 6.73
B7Q406	Hemelipoglyco-carrier protein	145.37	14.78, 2, 18, 56	1556, 177.5, 6.76
B7QGE3		91.39	5.36, 2, 1, 33	1325, 151.6, 6.77
B7PJC0		35.37	23.93, 1, 2, 12	117, 13.2, 8.29
B7PU24	Alpha-2-macroglobulin	20.16	13.80, 1, 6, 7	413, 44.7, 5.24
B7QMC8		10.01	3.11, 1, 4, 4	1092, 121.1, 5.73
B7P1G7	Angiotensin-converting enzyme	7.28	5.94, 2, 2, 3	320, 37.4, 6.13
B7PH04/A0A4D5RL95	Vitellogenin-b	5.78	7.97, 1, 2, 2	276, 31.7, 7.24
Fraction 2: No effect				
B7Q407	Heme lipoprotein	11.25	2.86, 3, 4, 5	1329, 152.5, 6.73
B7Q406	Hemelipoglyco-carrier protein	4.76	1.09, 1, 2, 2	1556, 177.5, 6.76
Fraction 3: Abnormal behavior pattern				
B7QM90/ A0A0K8RB81	Salivary gland metalloprotease	61.10	10.30, 2, 4, 26	437, 50.2, 7.49
B7QM92	Peptidase M12B domain-containing protein	50.88	7.58, 1, 3, 22	488, 55.7, 7.25
B7QM91	Secreted metalloprotease	15.82	7.94, 1, 4, 7	403, 45.9, 8.90
B7PST4	Serpin-4 precursor	12.66	7.51, 1, 3, 5	213, 23.6, 6.55
B7P904	Secreted protein	10.94	10.13, 1, 4, 5	306, 34.1, 6.02
B7PBG2	Actin	5.17	6.27, 1, 2, 2	335, 37.6, 5.55
Fraction 4: Hemorrhagic type allergic reactions and mortality				
B7QM92/A0A0K8RCY8	Peptidase M12B domain-containing protein. Putative metalloprotease	41.91	6.35, 2, 3, 16	488, 55.7, 7.25
B7P208/A0A0K8RKR7	Secreted protein-salivary antigen p23	12.00	11.59, 1, 2, 4	164, 18.3, 9.58
B7Q2B8	Metalloprotease	10.93	3.42, 2, 2, 4	468, 53.7, 6.58
Fraction 5: Abnormal or no feeding				
B7QKC1/ Q4PMH7	Anticoagulant Salp11-like	16.50	35.59, 1, 2, 5	59, 6.8, 4.22

**Table 2:** Gene Ontology annotations of tick saliva proteins associated with AGS

Tick proteins	Cellular component	Molecular function	Biological process
Alpha-macroglobulin	Extracellular region or secreted	Endopeptidase inhibitor activity	Complement activation
Angiotensin-converting enzyme Cofactor: Zn^2+^	Membrane	Carboxypeptidase activity Metal ion binding Metallopeptidase activity Peptidyl-dipeptidase activity	Regulation of inflammatory response, cytokine production, transmembrane transporter activity
Vitellogenin	Membrane	Glycosyltransferase activity Lipid transporter activity	Cellular response to heat, estradiol, insulin, polycyclic arene
Metalloprotease	Membrane	Metallopeptidase activity	Membrane protein ectodomain proteolysis
Actin	Cytoskeleton Nucleus	ATP binding Constituent of cytoskeleton	Mitotic cytokinesis Substantia nigra development Regulation of transmembrane transporter activity
Anticoagulant	Extracellular region and/or exosome	Unknown	Blood coagulation
Serpin	Extracellular region or secreted	Protein serine kinase activity Serine-type endopeptidase inhibitor activity	Negative regulation of endopeptidase activity, protein processing
Secreted protein B7P904	Secreted	Protein serine/threonine phosphatase activity	
Secreted protein B7P208-Salivary antigen p23	Secreted	Match to 3UV1_A Chain(A) PDB structure. Allergen from dust mite, *Dermatophagoides farinae* (DOI:10.2210/pdb3UV1/pdb) Bactericidal permeability-increasing protein	

### Quantitation of tick saliva proteins and α-Gal content

Protein and α-Gal content in tick saliva were determined in whole saliva and protein, non-protein, and deglycosylated fractions (Fig. 2A). The α-Gal levels were determined by an in-house enzyme-linked immunosorbent assay (ELISA) using tick saliva fractions in comparison with pig kidney (α-Gal-positive control) and human promyelocytic leukemia HL60 cells ATCC CCL-240 (α-Gal-negative control) (Fig. 2B) as described previously [22]. Tick saliva was diluted 1:1 in PBS and used to quantify α-Gal and protein content. Tick saliva, pig kidney, and HL60 protein concentrations were determined using a BCA Protein Assay Kit (Thermo Fisher Scientific) following the manufacturer’s recommendations. Briefly, ELISA plates were coated with 100 ng proteins per well from different samples in carbonate/bicarbonate buffer (Sigma-Aldrich), incubated overnight at 4 °C following five washes with PBS containing 0.05% Tween 20 (PBST), and unspecific unions blocked with 1% human serum albumin (HSA; Sigma-Aldrich). Anti-α-Gal epitope monoclonal antibodies (M86; Enzo Life Sciences Inc., Farmingdale, NY, USA) were added at 1:100 dilution in PBS and incubated for 1 h at 37 °C followed by four washes with PBST, and anti-mouse IgM (μ-chain-specific)-peroxidase antibodies produced in goat (Sigma-Aldrich) were added at 1:2000 dilution in PBS. The average value of the blanks (wells without sample proteins; *n* = 5) was subtracted from all reads, and the analysis was conducted using a calibration curve with 0.0 to 10.0 ng α-Gal (Galα1-3Gal-BSA, 3 atom spacer, product code NGP0203; Dextra, Shinfield, UK) and optical density (OD) values at 450 nm using Microsoft Excel for Mac (v. 16.26) to convert ELISA reader values to α-Gal content per sample (*R*^2^ = 0.96). Values for α-Gal content on each sample were represented as nanograms of α-Gal per microgram of proteins. As a control, wells coated with tick saliva protein fraction (*n* = 3) were incubated with secondary anti-mouse IgM-peroxidase antibodies alone, and α-Gal content values were below 0.0005 ng/µg proteins, thus ruling out non-specific reactions.

### Zebrafish

Wild-type adult (6–8-month-old) AB male and female zebrafish were provided by Dr. Juan Galcerán Sáez from the Instituto de Neurociencias (IN-CSIC-UMH, Sant Joan d'Alacant, Alicante, Spain) and certified by Biosait Europe S.L. (Barcelona, Spain; https://biosait.com) as free of major fish pathogens [24]. Zebrafish were maintained in a flow-through water system at 27 °C with a light/dark cycle of 14 h/10 h and were fed twice daily at 9:30 and 13:30 with dry fish feed (Premium food tropical fish, DAPC, Valladolid, Spain; 50–70 μg/fish). On day 2 and until the end of the experiment at day 8, fish were fed dog food (Classic Red, ACANA, Champion Petfoods LP, Edmonton, Canada; 150–200 μg/fish). The composition of fish feed (cereals, fish and fish byproducts, soya, yeast, crustaceans, and algae) and dog food (23% lamb meat meal, 22% steel-cut oats, 5% fresh ranch-raised beef, 5% fresh Yorkshire pork, 5% lamb fat, 4% raw grass-fed lamb, 2% whole oats, 2% fresh beef liver, 2% pork meat meal, 2% herring oil, 2% fresh pork liver, 1% fresh beef tripe, 0.1% freeze-dried beef liver, whole red lentils, whole green peas, whole green lentils, whole garbanzo beans, whole yellow peas, sun-cured alfalfa, lentil fiber, dried brown kelp, fresh pumpkin, fresh butternut squash, fresh parsnips, fresh green kale, fresh spinach, fresh carrots, fresh Red Delicious apples, fresh Bartlett pears, fresh cranberries, fresh blueberries, chicory root, turmeric root, milk thistle, burdock root, lavender, marshmallow root, and rosehips) were as used in previous zebrafish studies [24]. Zebrafish were euthanized by overdose of tricaine methane sulfonate (MS222, 200–300 mg/l) by prolonged immersion (https://citeseerx.ist.psu.edu/document?repid=rep1&type=pdf&doi=32ed904477ecfcc4b0ac4f7ece7483d888149694) [36].

### Characterization of anti-tick protein IgM antibody titers in zebrafish

Tick salivary gland protein extracts were prepared from salivary glands of *I. ricinus* eight female ticks-pool feeding for 5 days. Salivary glands were resuspended in 100 µl 1% Triton X-100-PBS solution and vortexed three times for 30 s. Then, the suspension was digested through a pellet pestle (DWK Life Sciences Kontes™ Pellet Pestle) and sonicated three times for 3 min. The BCA protein assay (Bio-Rad) was used for total protein quantification. For ELISA IgM titers quantification, high absorption capacity polystyrene microtiter plates were coated with 50 ng per well of tick saliva proteins in carbonate/bicarbonate buffer (Sigma-Aldrich). After overnight incubation at 4 °C, coated plates were washed once with 200 µl PBST (Sigma-Aldrich) and then blocked with 100 µl per well of 5% skim milk (Condalab, Madrid, Spain) in PBST (blocking solution) at room temperature (RT) with gentle shaking. Zebrafish serum samples from different groups of treatment were added at 1:100 dilution in blocking solution and incubated at 37 °C for 1 h. Plates were washed three times with PBST and 100 µl per well of specific rabbit anti-zebrafish IgM antibody diluted at 1:1000 in blocking solution. Plates were then incubated for 1 h at RT with gentle shaking. Plates were washed three times with PBST. A goat anti-rabbit IgG-peroxidase conjugate (Sigma-Aldrich) was added at 1:1000 and incubated for 1 h at RT with agitation. After three washes with 100 µl per well of PBST, 100 µl/well of TMB One Solution (Promega) was added and incubated for 15 min at RT in the dark. Finally, the reaction was stopped with 50 µl/well of 2 N H_2_SO_4_ and the OD at 450 nm was measured in a spectrophotometer (Multiskan, Thermo Fisher Scientific).

### Characterization of anti-α-Gal IgM antibody titers in zebrafish

The ELISA was conducted as for tick proteins, but plates were coated with 100 ng α-Gal (Galα1-3Gal-BSA, 3 atom spacer, approximately 1.82 × 10^20^ Gal epitopes/g [37]; product code NGP0203; Dextra, Shinfield, UK) per well in carbonate/bicarbonate buffer (Sigma-Aldrich), incubated overnight at 4 °C following five washes with PBST. Unspecific unions were blocked with 1% HSA (Sigma-Aldrich) for 1 h at RT. Serum peritoneal fluid samples were diluted (1:100, v/v) in blocking solution, followed by the addition of 100 μl/well and incubation for 1.5 h at 37 °C. Plates were washed three times with PBST, and 100 μl/well of rabbit anti-zebrafish IgM antibodies diluted (1:1,000, v/v) in blocking solution was added and incubated for 1 h at RT. Plates were washed with PBST, and goat anti-rabbit IgG-peroxidase conjugate (Sigma-Aldrich) diluted 1:3000 in blocking solution was added and incubated for 1 h at RT. After washes with PBST, 100 μl/well of TMB (Promega) was added and incubated for 15 min at RT. Reactions were stopped with 50 μl/well of 2N H_2_SO_4_, and the OD at 450 nm was measured in a spectrophotometer (Multiskan, Thermo Fisher Scientific). Only hemorrhagic type allergic reactions were associated with individual fishes treated with tick saliva, and thus a correlation analysis was conducted between anti-α-Gal IgM antibody titers and these signs in this group (*P* < 0.05; *n* = 6).

### Characterization of anti-glycan IgM antibody response in zebrafish

The glycochip array containing 378 glycans (20 µM) and 225 bacterial polysaccharides (2 µg/ml) was prepared as previously described (Semiotik LLC, Russia) [38]. Pooled sera obtained from a previous experiment [39] of 10 zebrafish for each group immunized by immersion with bovine serum albumin (BSA) coated with α-Gal (α-Gal; Dextra, Shinfield, UK) and PBS-treated control were diluted 1:10 in PBST (Sigma-Aldrich) and incubated with glycochip arrays overnight at 4 °C in a humidified chamber. After thorough washing with PBST to remove the proteins, glycochips were incubated with IgGs from rabbits immunized with zebrafish IgM diluted 1:1000 in PBST for 45 min at 20 °C. Then, glycochips were washed with PBST and incubated with goat anti-rabbit IgG (H + L)-Alexa Fluor 532 nm (Thermo Fisher Scientific) diluted 1:1000 in PBST at 20 °C for 1 h. Fluorescence signal intensity corresponding to the antibodies bound to printed glycans was measured with a GenePix 4100A fluorescence scanner (Molecular Devices, San Jose, CA, USA) at 500 PMT and a resolution of 10 µm. The images were processed using ScanArray Express 4.0 (fixed circle method) and then by Microsoft Excel software. Six spots represent each oligosaccharide or polysaccharide on the array, and data are reported as median relative fluorescence units (RFU) of replicates, given as a percentage ratio of maximum RFU on the chip (normRFU). The normRFU above 10% was considered significant (Additional file 2: Dataset S2).

### Statistical analyses

The incidence of allergic reactions, abnormal behavior and feeding patterns, and mortality in zebrafish were compared between treatments by one-way analysis of variance (ANOVA) test with post hoc Tukey honestly significant difference (HSD) test (*P* < 0.05; https://astatsa.com/OneWay_Anova_with_TukeyHSD/). Anti-tick proteins and anti-α-Gal IgM antibody titers (OD at 450 nm) in zebrafish were compared between treatments by one-way ANOVA test with Bonferroni–Holm multiple comparisons with only pairs relative to PBS simultaneously compared (*P* < 0.05; *n* = 6–10 biological replicates; https://astatsa.com/OneWay_Anova_with_TukeyHSD/).

## Results

### Characterization of protein and α-Gal content in tick saliva fractions and antibody response in zebrafish

Protein content varied between different tick saliva fractions with the lowest concentration in SDS-PAGE protein fractions 1–5 (Fig. 2A). Protein and α-Gal content was higher in whole tick saliva and protein fractions when compared with non-protein and deglycosylated components (Fig. 2B).

**Fig. 3:**
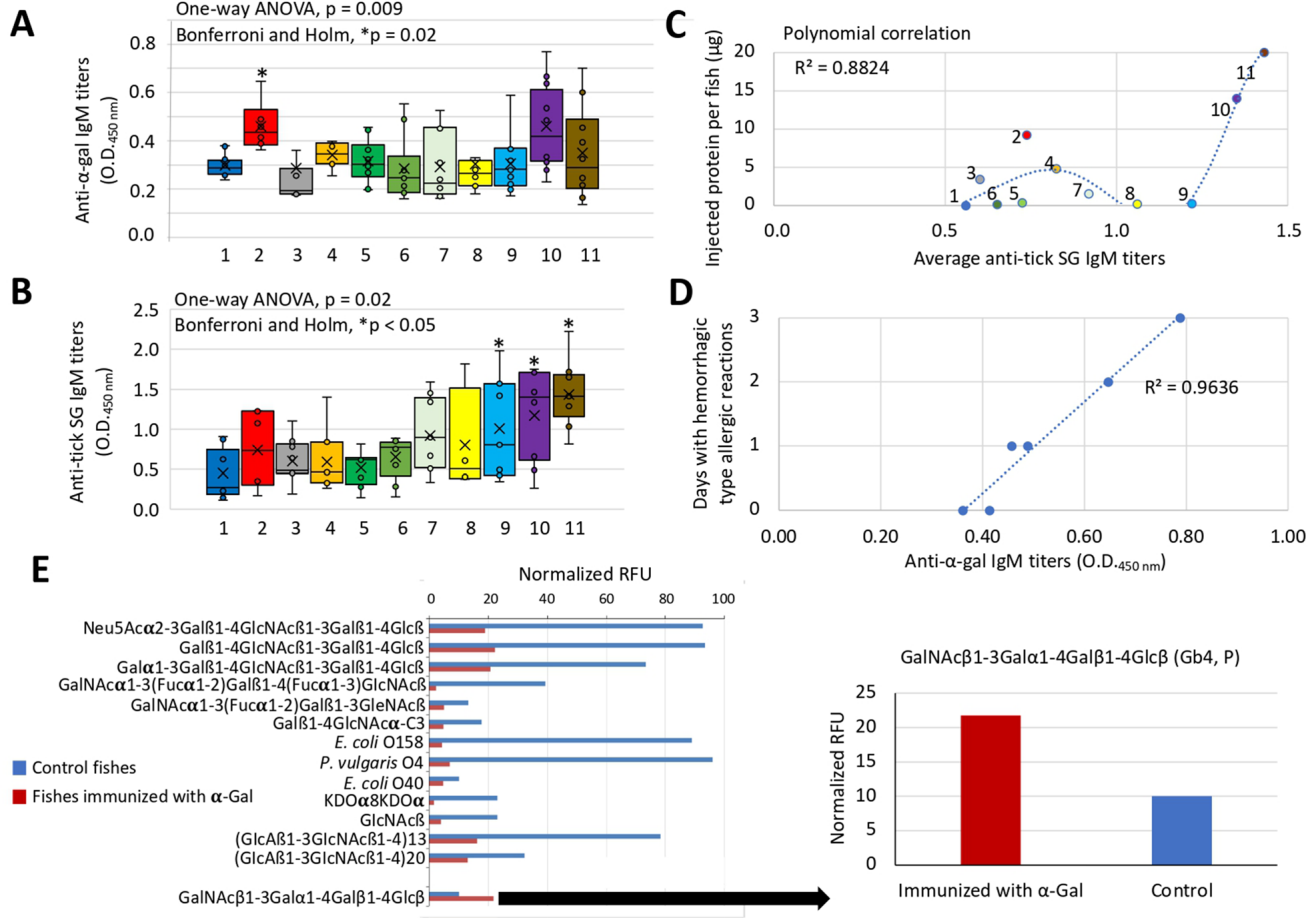
Antibody response in zebrafish treated with tick saliva components. After fish euthanasia, serum was collected from each animal to determine IgM antibody titers against **A** α-Gal and **B** tick proteins. Results were compared between treatments by one-way ANOVA test with Bonferroni–Holm multiple comparisons with only pairs relative to PBS simultaneously compared (*P* < 0.05; *n* = 6–10 biological replicates). **C** Polynomial correlation analysis between the amount of protein injected per fish and average anti-tick salivary gland IgM titers (*R*^2^ = 0.9). **D** Correlation analysis between hemorrhagic type allergic reactions and anti-α-Gal IgM antibody titers in fishes treated with tick saliva (*R*^2^ = 0.9, *P* < 0.001; *n* = 6). **E** Results of the anti-glycan IgM antibody response in zebrafish. Most of the reactive antibodies with significant differences were lower in response to α-Gal immunization, and the only glycan with higher RFU in α-Gal-immunized zebrafish was P antigen, Gb4 (https://www.omim.org/entry/615021). The glycochip array data and analysis are provided in full in Additional file 2: Dataset S2. Group numbers in panels **A**, **B**, and **C** correspond to treatments with PBS (1), saliva (2), saliva non-protein fraction (3), saliva protein fraction (4), protein fraction 1 (5), protein fraction 2 (6), protein fraction 3 (7), protein fraction 4 (8), protein fraction 5 (9), deglycosylated saliva (10), and deglycosylase plus buffer (11)

The anti-α-Gal IgM antibody titers were significantly higher only in zebrafish treated with tick saliva when compared with PBS-treated controls (*P* < 0.05; Fig. 3A). The results for tick saliva were in accordance with α-Gal content in this fraction (Fig. 2B). However, other unknown factors affected the anti-α-Gal IgM antibody titers in zebrafish treated with saliva protein fraction as the α-Gal content was relatively high in this fraction (Fig. 2B). Regarding the anti-tick salivary gland IgM antibodies, zebrafish treated with protein fraction 5, deglycosylated saliva and deglycosylase with buffer showed significantly higher antibody titers than PBS-treated control (*P* < 0.05; Fig. 3B). These results suggest that multiple factors affect antibody response to tick salivary gland biomolecules and agree with the polynomial correlation that was obtained between the amount of protein injected per fish and average anti-tick salivary gland IgM titers (*R*^2^ = 0.9; Fig. 3C). Furthermore, two protein components in the EDEGLY enzymatic protein deglycosylation kit (Merck & Co., Inc.), peptidylglycine monooxygenase (PNGase F) and glycogen debranching enzyme (β(1-4)-galactosidase) are highly conserved and present in *I. ricinus* ticks (A0A0K8R5I7 and A0A147BME2, respectively), which probably explains the high antibody titers in fish treated with deglycosylated saliva containing PNGase F and deglycosylases in buffer fractions (Fig. 3B). A positive correlation was obtained between hemorrhagic type allergic reactions and anti-α-Gal IgM antibody titers in zebrafish treated with tick saliva (*P* < 0.001; Fig. 3D).

The results of the anti-glycan IgM antibody response in zebrafish showed that most of the reactive antibodies with significant differences were lower in response to α-Gal immunization (Fig. 3E; Additional file 2: Dataset S2). The only glycan with higher RFU in α-Gal-immunized zebrafish was GalNAcβ1-3Galα1-4Galβ1-4Glcβ (also known as globoside-4, Gb4 or P antigen), an antigen of the human GLOB blood group system (Fig. 3D; Additional file 2: Dataset S2).

### Characterization of tick saliva components associated with allergic reactions to mammalian meat consumption in the zebrafish model of AGS

**Fig. 4:**
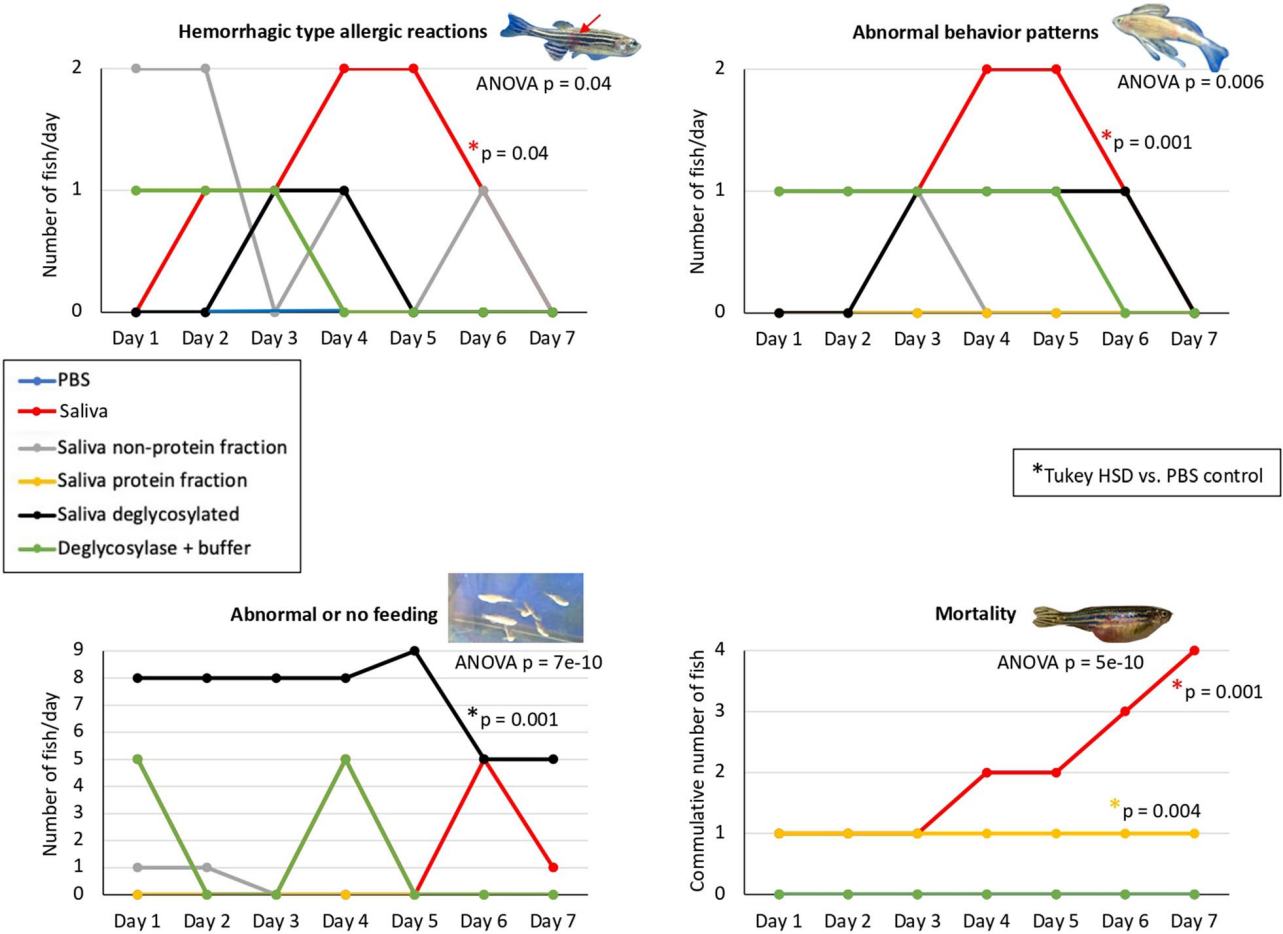
Allergic reaction of zebrafish to whole tick saliva and components. After treatment with whole tick saliva and saliva non-protein, protein, and deglycosylated components at days 0 and 3, with PBS and buffer with deglycosylase as controls, zebrafish were examined and the incidence of hemorrhagic type allergic reactions, abnormal behavior and feeding patterns, and mortality were compared between treatments by one-way ANOVA test with post hoc Tukey HSD test (*P* ≤ 0.04; *n* = 6–10 biological replicates). Significant differences between treatments and PBS control are shown with post hoc Tukey HSD *P*-values

**Fig. 5:**
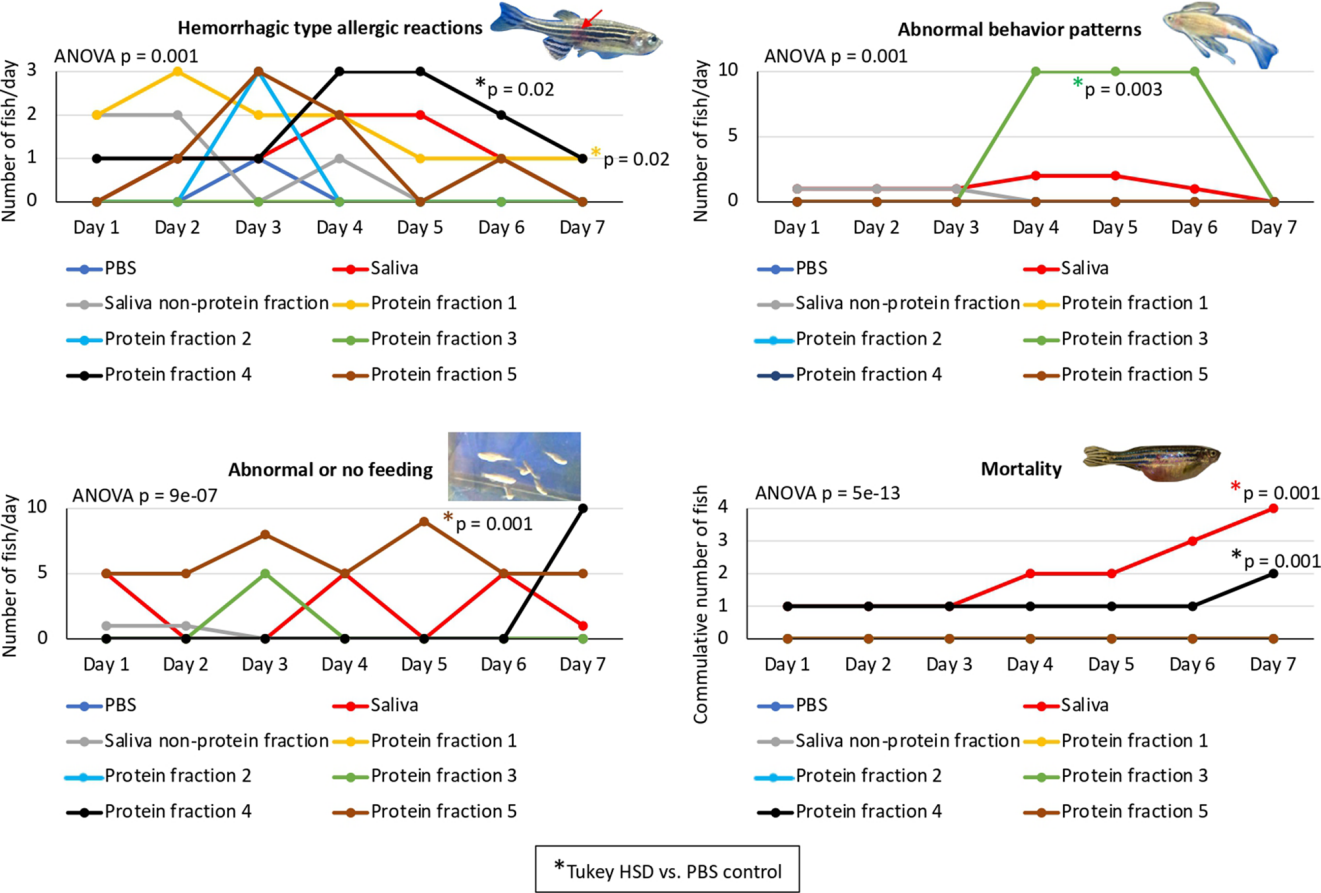
Allergic reaction of zebrafish to whole tick saliva and protein fractions. After treatment at days 0 and 3 with tick whole saliva and different protein fractions (1–5) combined with non-protein fraction using PBS as control, zebrafish were examined and the incidence of hemorrhagic type allergic reactions, abnormal behavior and feeding patterns, and mortality were compared between treatments by one-way ANOVA test with post hoc Tukey HSD test (*P* ≤ 0.02; *n* = 6–10 biological replicates). Significant differences between treatments and control are shown with post hoc Tukey HSD *P*-values

Treatment with tick saliva after feeding on dog food resulted in a significantly higher incidence of hemorrhagic type allergic reactions, abnormal behavior patterns, and mortality when compared with PBS-treated control (*P* ≤ 0.04; Fig. 4) and was associated with higher anti-α-Gal IgM antibody titers at least for hemorrhagic type allergic reactions (Fig. 3D). The signs seen on fish treated with different tick salivary components on day 1 before dog food challenge may be due to injection and/or direct toxic effect of tick saliva on the fish model and not saliva–mammalian meat-associated reactions (Figs. 4 and 5). Nevertheless, result analyses were based on statistically significant differences when compared with PBS-treated control (Figs. 4 and 5).

Only treatment with deglycosylated saliva, but not deglycosylases with buffer, significantly altered feeding when compared with control (*P* = 0.001; Fig. 4). Then, treatment with five saliva protein fractions separated by SDS-PAGE in combination with the non-protein fraction to mimic saliva were compared (Fig. 4). The incidence of hemorrhagic type allergic reactions was significantly higher in saliva protein fractions 1 and 4 treated groups when compared with PBS control (*P* = 0.02). Abnormal behavior and feeding were associated with treatment using saliva protein fractions 3 (*P* = 0.003) and 5 (*P* = 0.001), respectively. Significant mortality was caused by treatment with whole saliva and protein fraction 4 (*P* = 0.001). However, it should be considered that the fractionation of the salivary proteins by SDS-PAGE may affect in different degrees the structure and activity of enzymes, which may translate into differences in signs produced in zebrafish after treatment with tick saliva protein fractions.

**Fig. 6:**
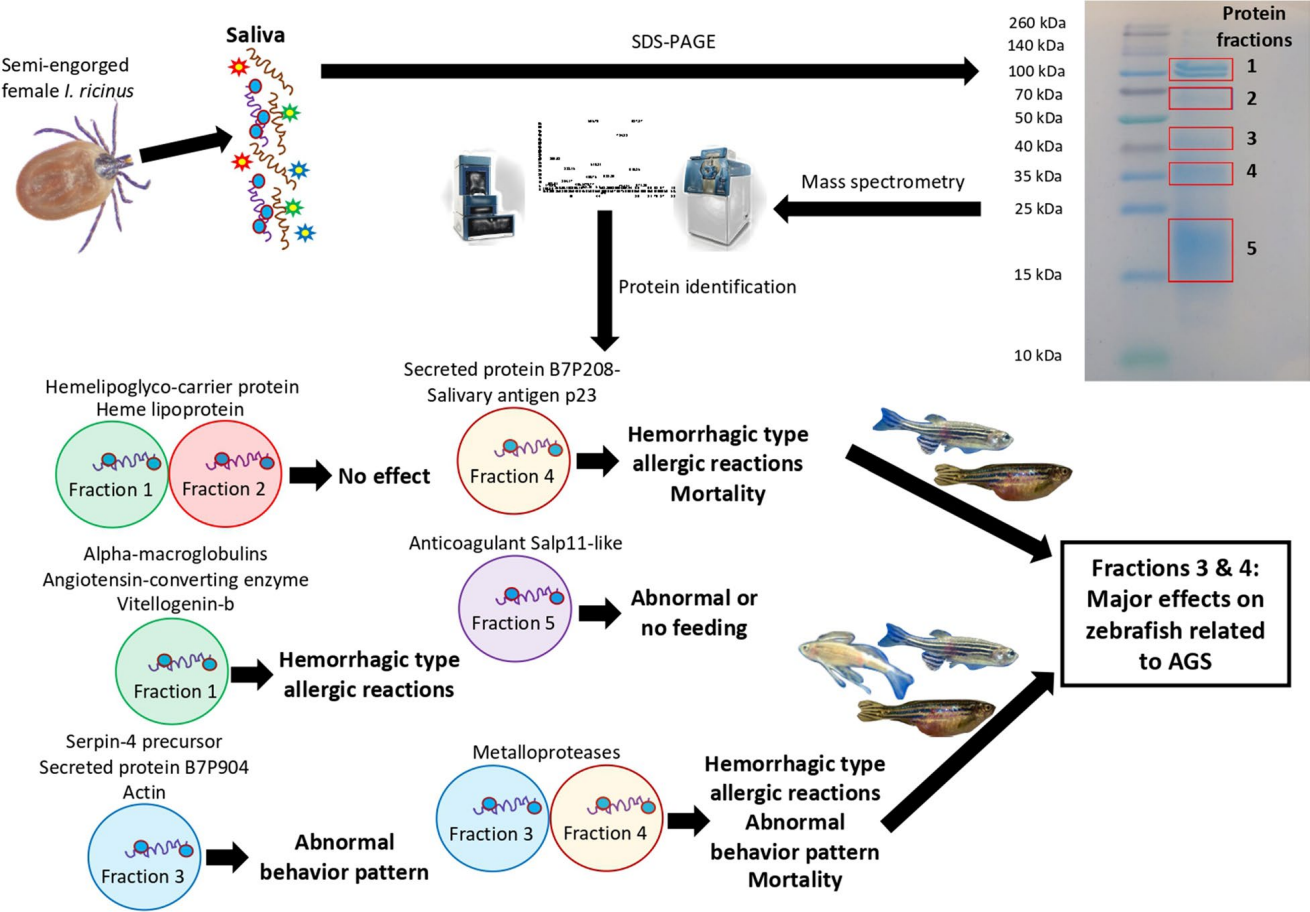
Identification and functional association with the alpha-Gal syndrome (AGS) of proteins in tick saliva. Tick saliva proteins were fractionated by SDS-PAGE, and five fractions were extracted and proteins identified by mass spectrometry analysis. The results (Additional file 1: Dataset S1) were then associated with their effect on zebrafish allergic reaction to treatment with different protein fractions to identify those associated with major effects on zebrafish related to AGS

The identification of proteins in the five saliva fractions separated by SDS-PAGE was then approached using mass spectrometry analysis (Fig. 6, Table 1, Additional file 1: Dataset S1). For functional implication in AGS, identified proteins were then associated with their effect on zebrafish allergic reaction to treatment with different protein fractions (Fig. 5) to identify those associated with major effects on zebrafish related to AGS (Fig. 6, Table 1, Additional file 1: Dataset S1). Major effects were related to secreted protein B7P208-salivary antigen p23 (predicted allergen; Additional file 1: Dataset S1) in protein fraction 4 associated with hemorrhagic type allergic reactions and mortality, and metalloproteases identified in fractions 3 and 4 and associated with hemorrhagic type allergic reactions, abnormal behavior patterns, and mortality (Fig. 6, Table 1). Other tick salivary proteins were identified as associated with hemorrhagic type allergic reactions (fraction 1: alpha-macroglobulins, angiotensin-converting enzyme, vitellogenin-b), abnormal behavior pattern (fraction 3: serpin-4 precursor, secreted protein B7P904, actin), and abnormal or no feeding (fraction 5: anticoagulant salp11-like) (Fig. 6, Table 1).

Gene Ontology annotation of identified tick saliva proteins associated with AGS in zebrafish showed as expected that most proteins were extracellular or secreted and associated with multiple metabolic processes (Table 2). Some of the identified tick proteins associated with AGS in the zebrafish model were previously reported as recognized by patients IgE but not by healthy individuals or using an allergenomics approach and reactive or not with α-Gal (Table 3). Other proteins were associated with allergenic compounds or acquired resistance to *I. scapularis* (Table 3).

**Table 3:** Tick salivary proteins associated with allergic reactions, AGS, or acquired resistance to tick infestations

Tick proteins	Signs associated with AGS in zebrafish model	Association with allergy, AGS in humans	Association with acquired resistance to tick infestations	α-Gal-positive	References
Alpha-macroglobulin	Hemorrhagic type allergic reactions	Yes	Yes	Yes	[13, 34, 41, 54, 55]
Vitellogenin	Hemorrhagic type allergic reactions	Yes	No	Yes	[13, 55]
Metalloprotease	Hemorrhagic type allergic reactions, abnormal behavior pattern, and mortality	Yes	No	No	[55]
Actin	Abnormal behavior pattern	Yes	No	No	[13, 55]
Anticoagulant	Abnormal or no feeding	No	Yes	Unknown	[34, 54]
Serpin	Abnormal behavior pattern	No	Yes	Yes	[34, 54, 55]
Secreted protein B7P208-Salivary antigen p23 (allergen)	Hemorrhagic type allergic reactions and mortality	Yes	No	Unknown	[55, 56]

## Discussion

As previously reported in the proposed zebrafish model of AGS [24], treatment with tick saliva resulted in a significantly higher incidence of hemorrhagic type allergic reactions, abnormal behavior patterns, and mortality, with a positive correlation between hemorrhagic type allergic reactions and anti-α-Gal IgM antibody titers. These results provided additional support for the use of zebrafish as an AGS animal model.

The results obtained here support the contention that exposure to tick saliva is associated with these signs, which have been described in cases with AGS [1, 2, 8, 22, 31, 40–45]. However, it is important to consider the potential effect of pilocarpine used for tick saliva extraction and which may be mixed in tick salivary components. Pilocarpine treatment in zebrafish has been shown to cause behavioral and biochemical alterations in chronic seizure-like conditions after repeated treatments [46]. Some of the abnormal behavior patterns observed in zebrafish treated with tick saliva and saliva protein fraction 3 may be associated with pilocarpine residues in these treatments. Considering this possibility, we compared the dose of pilocarpine causing seizure-like conditions in zebrafish (> 200 mg/kg) [46] with that used in our experiment. Considering that we treated ticks with 5 μl of a 2% (w/v) solution, equivalent to 2 g/100 ml or 20 mg/ml, and pilocarpine recovered in *I. scapularis* saliva is 11.5 μg/μl when treated with 2 μl of 50 mg/ml solution [47], the estimate in our experiment is also of 11.5 μg/μl pilocarpine in *I. ricinus* saliva. In the experiment reported here, 1 μl saliva was injected per treatment in zebrafish, equivalent to 33 mg/kg of pilocarpine, which is one sixth the amount causing seizure-like conditions in zebrafish [46]. Therefore, it is unlikely that the signs observed here in zebrafish in response to tick saliva components are associated with pilocarpine residues. Furthermore, toll-like receptor 4 (*tlr4*) messenger RNA (mRNA) levels were reported to increase in response to high pilocarpine doses in zebrafish with seizure-like conditions [46], but in zebrafish treated with tick saliva, *tlr4* upregulation was observed only in the intestine of animals fed α-Gal-positive dog food and not those with α-Gal-negative fish feed [24].

Regarding tick saliva components, the results suggested a role for saliva non-protein biomolecules in the production of hemorrhagic type allergic reactions. However, glycosylation of salivary proteins appears to confer protection from abnormal or no feeding behavior, which correlates with higher anti-α-Gal IgM antibody titers in some zebrafish treated with deglycosylated saliva (Fig. 3A). In accordance with these results, Park et al. [48] proposed that glycan α-Gal serves as a molecular mimic of bioactive proteins during tick feeding on mammalian hosts but contributes as a sensitizer to allergic reactions associated with AGS in an atypical human host and in the zebrafish model.

The allergic response to the combination of tick saliva proteins with the non-protein fraction supports that both protein and non-protein components are involved in the modulation of the host immune response that may be associated with AGS. As reported in this study, some of the tick proteins associated with allergic reactions (i.e., metalloproteases) do not contain α-Gal modifications but may be associated with allergy/AGS in humans through activation of immune-related mechanisms (Table 3).

As previously reported in chickens [49], and observed here in zebrafish, immunization with α-Gal-containing biomolecules from tick saliva may reduce the natural humoral immune response that may affect reactions to tick bites. Furthermore, IgM and IgG antibody levels in response to the P antigen, Gb4, a prominent glycosphingolipid on human erythrocytes [50, 51], increased in α-Gal-immunized zebrafish, which may be associated with hemolytic transfusion reactions and paroxysmal cold hemoglobinuria [52, 53].

## Conclusions

The limitations of the study to be considered include (1) the need to provide immune- and allergic-related biomarkers to support the response to tick saliva biomolecules in the zebrafish model, (2) the need to correlate at the molecular level the response in zebrafish treated with tick saliva components and fed mammalian meat with those reported in patients with AGS, (3) the low incidence of allergic reactions in zebrafish and the possibility that the presence of α-Gal in tick saliva might be important for α-Gal sensitization and allergic symptoms in the zebrafish model of AGS, (4) the wide inter- and intrapersonal variability in AGS symptomatology that may also be represented in the zebrafish model, (5) the fact that proteins > 50 kDa were removed from the deglycosylated fraction, (f) considering in future experiments saliva protein fractionation alternatives to SDS-PAGE approaches to reduce the effect on protein structure and activity, and (g) including control treatments with tick saliva unrelated proteins.

Despite these limitations, the results provide new insights to support the hypothesis that tick saliva biomolecules with and without α-Gal modifications are involved in modulating human immune response against this carbohydrate. The next step to advance in the diagnosis, treatment, and prevention of AGS is deciphering the immune-related mechanisms activated in response to these tick saliva components. These studies will be conducted by analysis of human sera for IgE to these proteins and using omics approaches in intestine samples collected from zebrafish in this study.

### Supplementary Information

**Additional file 1: Dataset S1.** Identification and characterization of tick saliva proteins in SDS-PAGE fractions 1–5 by mass spectrometry and association with allergic reactions in zebrafish.

**Additional file 2: Dataset S2.** Glycochip data analysis.

## Data Availability

The data presented in this study are disclosed in the paper and Supplementary information.

## Abbreviations

AGS: Alpha-Gal syndrome
α-Gal: Galactose-alpha-1,3-galactose
amu: Atomic mass units
BCA: Bicinchoninic acid
ELISA: Enzyme-linked immunosorbent assay
ESI: Electrospray ionization
FDR: False discovery rate
HSD: Honestly significant difference
Ig: Immunoglobulin
OD: Optical density
PBS: Phosphate-buffered saline
PBST: PBS/0.05% Tween 20
RFU: Relative fluorescence units
RP-LC–MS/MS: Reversed-phase liquid chromatography coupled to mass spectrometry
SDS-PAGE: Sodium dodecyl sulfate polyacrylamide gel electrophoresis
RT: Room temperature
